# Chronic but not acute nicotine treatment ameliorates acute inflammation‐induced working memory impairment by increasing CRTC1 and HCN2 in adult male mice

**DOI:** 10.1111/cns.14627

**Published:** 2024-02-14

**Authors:** Xiaona Wang, Qian Wang, Min Song, Yihui Wang, Xianzhi Shen, Yanyun Sun, Chun Guo, Panpan Geng, Chaolin Ma, Xinchun Jin

**Affiliations:** ^1^ School of Life Science Nanchang University Nanchang China; ^2^ Institute of Biomedical Innovation, Jiangxi Medical College Nanchang University Nanchang China; ^3^ Department of Histology and Embryology, School of Basic Medical Sciences, Advanced Innovation Center for Human Brain Protection, Beijing Key Laboratory of Cancer Invasion and Metastasis Research Capital Medical University Beijing China; ^4^ Institute of Neuroscience The Second The Second Affiliated Hospital of Soochow University Suzhou China; ^5^ School of Biosciences University of Sheffield Sheffield UK

**Keywords:** CRTC1, HCN2, lipopolysaccharide, mice, nicotine, working memory

## Abstract

**Background:**

Systemic inflammation in which lipopolysaccharide (LPS) is released into circulation can cause cognitive dysfunction and we have previously shown that LPS impaired working memory (WM) which refers to the ability to guide incoming behavior by retrieving recently acquired information. However, the mechanism is not very clear, and currently, there is no approved strategy to improve inflammation‐induced WM deficit. Notably, epidemiological studies have demonstrated a lower occurrence rate of inflammatory‐related diseases in smoking patients, suggesting that inflammation‐induced WM impairment may be improved by nicotine treatment. Here, our object is to investigate the effect and potential mechanisms of acute and chronic nicotine treatment on LPS‐produced WM deficiency.

**Methods:**

Delayed alternation T‐maze task (DAT) was applied for evaluating WM which includes both the short‐term information storage and the ability to correct errors in adult male mice. Immunofluorescence staining and immunoblotting were used for assessing the levels and distribution of CREB‐regulated transcription coactivator 1 (CRTC1) and hyperpolarization‐activated cation channels 2 (HCN2) in the medial prefrontal cortex (mPFC) and hippocampus. Quantitative PCR and ELISA were employed for analyzing the mRNA and protein levels of TNF‐α and IL‐1β.

**Results:**

Our results revealed that administration of LPS (i.p.) at a dose of 0.5 mg/kg significantly produced WM impairment in the DAT task accompanied by an increase in IL‐1β and TNF‐α expression in the mPFC. Moreover, intra‐mPFC infusion of IL‐1Ra, an IL‐1 antagonist, markedly alleviated LPS‐induced WM deficiency. More important, chronic (2 weeks) but not acute nicotine (0.2 mg/kg, subcutaneous) treatment significantly alleviated LPS‐induced WM deficiency by upregulating CRTC1 and HCN2. Of note, intra‐mPFC infusion of HCN blocker ZD7288 produced significant WM deficiency.

**Conclusions:**

In summary, in this study, we show that chronic nicotine treatment ameliorates acute inflammation‐induced working memory deficiency by increasing CRTC1 and HCN2 in adult male mice.

## Graphical abstract

LPS significantly induced WM deficit accompanied by the increase in IL‐1β and TNF‐α in mPFC. Nicotine treatment significantly alleviated LPS‐induced WM deficit by upregulating CREB‐regulated transcription coactivator 1 (CRTC1) and hyperpolarization‐activated cation channels 2 (HCN2).

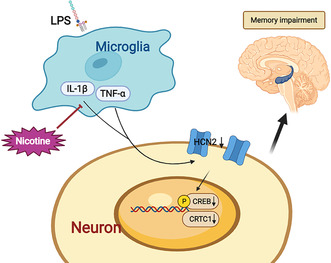

## 1 INTRODUCTION

Prefrontal cortex (PFC) and hippocampus‐dependent working memory (WM) refer to the ability to possess relevant information online in coordinating goal‐directed behavior.^1^ WM deficiency would disrupt the capacity to instruct incoming behavior through retrieval of recently acquired information. It could be disrupted under various pathological conditions such as Alzheimer's disease,^2^ schizophrenia,^3^ and stress.^4^

Systemic inflammation, which releases lipopolysaccharide (LPS) into circulation,^5^ can cause emotional and cognitive dysfunction through the release of a substantial quantity of inflammatory factors and the activation of immune cells.^6^ We have shown that acute LPS treatment induced significant WM deficiency in the DAT task, accompanied by decreased synaptophysin levels.^7^ However, it is not known how LPS induced WM deficiency and there is currently no effective strategy for inflammation‐induced WM deficiency.

CREB‐regulated transcription coactivator 1 (CRTC1), a key regulator of gene transcription driven by the cAMP response element (CRE),^8^ plays essential roles in memory consolidation^9^ and reconsolidation.^10^ CRTC1 dysfunction is associated with memory deficiency caused by ischemic stroke,^11^ Alzheimer's disease,^12^ and LPS.^13^ Nevertheless, it remains uncertain whether CRTC1 is implicated in inflammation‐induced WM damage. In addition, LPS has been demonstrated to regulate the activity of hyperpolarization‐activated cation channels (HCN),^14^ a channel that plays important roles in synaptic plasticity^15^ and memory.^16^ For example, HCN is involved in memory deficiency induced by chronic cerebral hypoperfusion^17^, ^18^ or chronic morphine exposure.^19^ In addition, it has been shown that inhibition of cAMP‐HCN in PFC enhanced WM.^20^ However, it is unclear whether HCN is related to inflammation‐produced WM deficiency.

Although the detrimental health consequences of tobacco smoking are widely reported, epidemiological studies have also revealed a lower occurrence rate of several neurodegenerative diseases^21^ and inflammatory diseases^22^ in smoking patients. In addition, chronic smoking, but not acute nicotine treatment, affected neural correlates of WM.^23^ Furthermore, chronic nicotine treatment reduced sepsis‐produced oxidative damage of multiple organs^24^ and we have demonstrated that acute nicotine administration (0.2 mg/kg) improved the performance of spatial working memory in adult male rats in DAT task.^25^ Therefore, in this study, we examined the effects of both acute and chronic nicotine treatments on WM deficiency induced by acute LPS treatment using the DAT task and showed that chronic (2 weeks) but not acute nicotine (0.2 mg/kg) treatment improved LPS‐produced WM deficiency through upregulating CRTC1 and HCN2 in adult male mice.

## 2 MATERIALS AND METHODS

### 2.1 Subjects

Male C57BL/6 mice (8–10 weeks) were obtained from SLAC Laboratory Animal (Shanghai, China). They were raised in specific pathogen‐free (SPF)‐level environment with constant temperature (23 ± 1°C) and were subjected to a restricted diet for 1 week before the commencement of the experiment to maintain 85% of their body weight. All experimental designs were approved by the Animal Care Committee of Soochow University and were carried out following the NIH Guide for the Care and Use of Laboratory Animals. All designs followed the principle to reduce and minimize the number of animals and their suffering.

### 2.2 Surgery

The mice were anesthetized by intraperitoneal (i.p.) injection of pentobarbital sodium (5 mL/kg), followed by the bilateral implantation of 23‐gauge stainless‐steel guide cannula. The mPFC coordinates were obtained from the Paxinos and Watson 1986 atlas (anterior–posterior, +1.8 mm; medial–lateral, ±0.45 mm; and dorsal–ventral, 2.1 mm from skull surface).^26^ Sterile stainless‐steel screws and dental cement were employed to anchor the guide cannula to the cranium. To prevent infection, a sterile dummy cannula was inserted into the guide cannula to maintain sterility. Subsequently, following surgery, the mice underwent a 5‐ to 7‐day recovery period.

### 2.3 Drugs and drug administration

Nicotine hydrogen tartrate (Sigma Chemical Company, St. Louis, MO, USA) dissolved in saline (pH = 7.4) was injected subcutaneously at 0.2 mg/kg. Nicotine tartrate was used as the term to represent all nicotine doses throughout the text.

Lipopolysaccharide (LPS, Sigma, St. Louis, MO, USA) was dissolved in sterile saline and intraperitoneal injected for 4 h before testing with a dose of 0.5 mg/kg which can produce WM deficiency in DAT task in T maze.^7^

HCN channel blocker ZD7288 (Sigma, St. Louis, MO, USA) was intra‐PFC infused with 1.5 μg/μL, or 15 μg/μL 15 min before testing.^27^

IL‐1 receptor antagonist IL‐1Ra (Techno Gene, 1 μg/1 μL/day, dissolved in saline) was intra‐PFC infused through a guide cannula 5 h before testing.^28^

### 2.4 Behavioral experiments

#### 2.4.1 Delayed alternation T‐maze task

There were three free shuttle arms in the mouse T‐maze: an initiation arm (30 × 10 × 30 cm) and two detection arms (35 × 10 × 30 cm each). The behavioral test was done between 1:00 p.m. and 5:00 p.m.

As we described previously, mice were trained using the delayed alternation task (DAT).^7^ The daily food of mice was limited, but water was sufficient. First, the mice had to acclimatize in the T‐maze for 2 days, and the trials began on the third day. Each daily session began with one initial trial, followed by nine normal trials. Briefly, mice were trained to visit the two detection arms in an alternating pattern, receiving a reward for each correct choice. In the initial trial, there was food at the end of both arms so that the mice could achieve food by choosing either arm. Mice were placed in the starting zone, the gate was opened, and after the mice reached the end of either arm to obtain reward, mice were replaced into the starting zone again. During normal trials, mice only received the reward when they entered the opposite arm. Otherwise, the mice had to return to the starting zone to restart the train trial. The mice can proceed to the next normal trial only when they can successfully get the reward. The T‐maze would be cleaned with alcohol to remove odors between sessions, at a 10‐s interval.

T‐maze training lasted for about 1 week. The formal trial began when the mice achieved an 80% success rate for 3 consecutive days (the correct choice accounted for a percentage of all choices, and the correct choice was always 9). Four hours after LPS treatment, each mouse was tested 10 times and the numbers of two types of errors were recorded: win‐shift failure (incorrect choice following a previous correct choice) and lose‐shift failure (incorrect choice following a previous incorrect choice). Specifically, the number of win‐shift failures reflected the spatial working memory of mice, and the number of lose‐shift failures reflected the error correction ability of mice.^7^

#### 2.4.2 Measurement of locomotor activity

The behavior response of mice in the T‐maze during the WM test was reported by a camera and a computer connected to it with Anymaze software (Shanghai Xinruan Information Technology Co. Ltd). Based on the time taken by the mouse and the distance traveled, speed was calculated.

#### 2.4.3 Experimental procedures

##### Experimental 1

**FIGURE 1:**
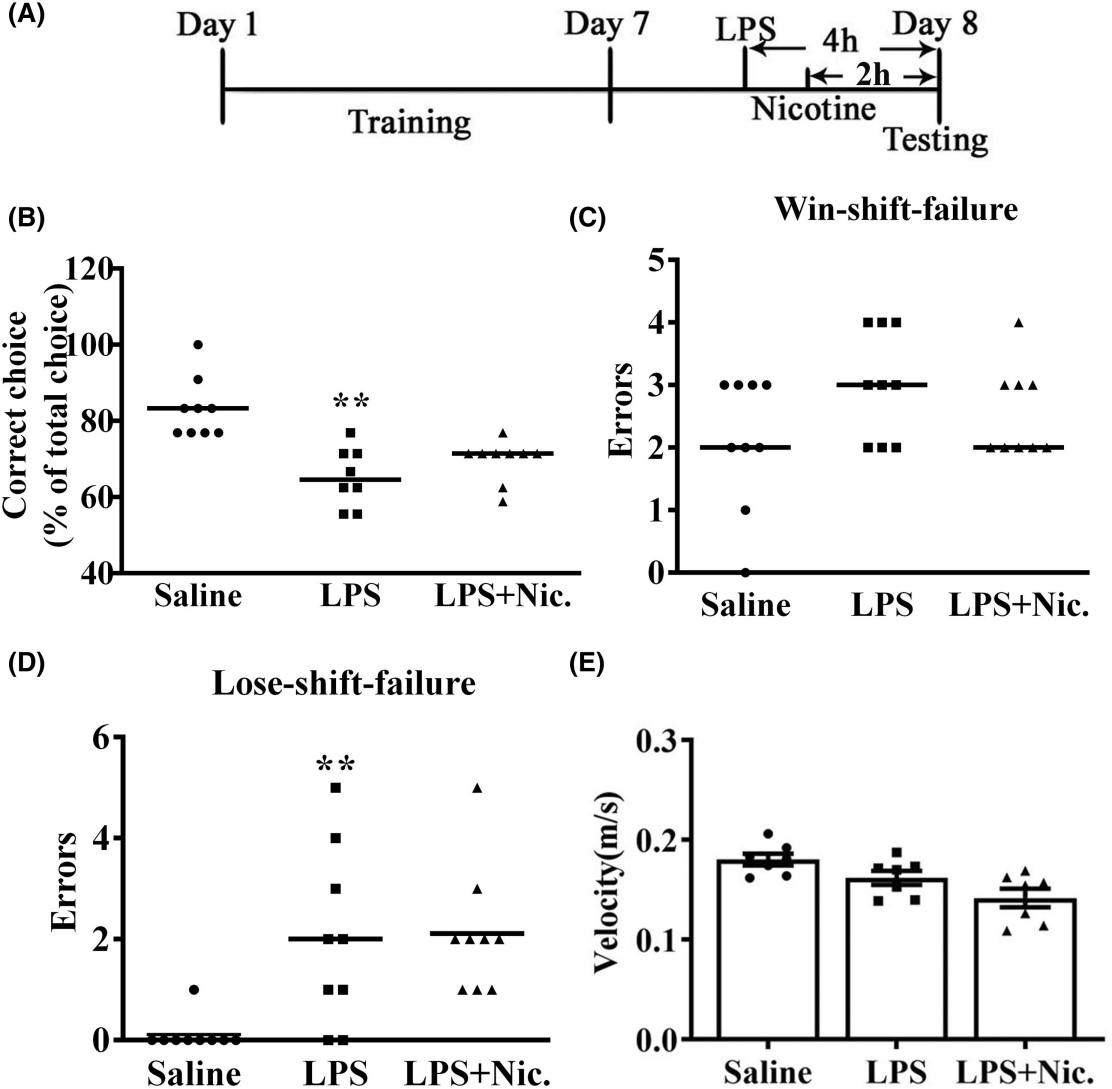
Effect of acute nicotine treatment on LPS‐induced WM deficit in DAT task. (A) The diagram illustrated the procedure in the experiment. (B) LPS treatment showed significant downregulation of the correct choice rate of mice in the DAT task (**p < 0.01 vs. the saline group), and acute nicotine treatment failed to dramatically improve the reduction of the correct choice (*p* > 0.05). (C, D) LPS treatment had no notable impact on the win‐shift failure (*p* > 0.05), but significantly increased lose‐shift failure (**p < 0.01 vs. the Saline group), and acute nicotine treatment did not significantly improve LPS's effect (*p* > 0.05; *n* = 9). (E) LPS and acute nicotine treatment had no significant impact on motor function (*p* > 0.05, *n* = 7).

This test aimed to examine the impact of acute nicotine treatment on LPS‐produced WM deficiency. Mice were randomly divided into three groups: saline, saline+LPS (0.5 mg/kg, i.p.), and nicotine (0.2 mg/kg, s.c.) + LPS (0.5 mg/kg, i.p.) (see Figure 1A for detailed experimental procedures).

##### Experimental 2

**FIGURE 2:**
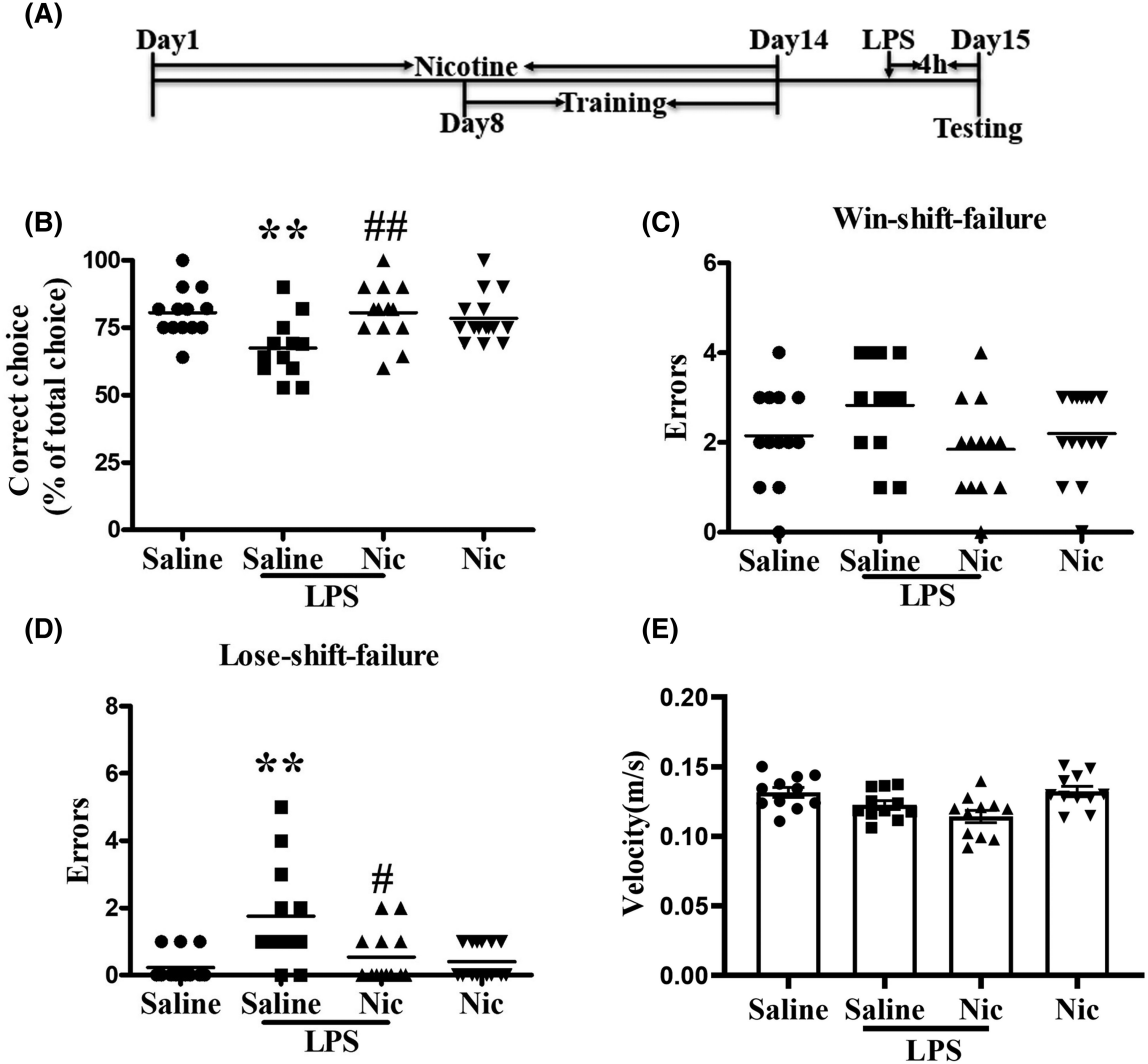
Effect of chronic nicotine treatment on LPS‐induced WM deficit in DAT task as well as the levels of pro‐inflammatory factors IL‐1β and TNF‐α in the PFC. (A) Diagram of the experimental procedure. (B) LPS treatment markedly reduced the correct choice rate of mice in DAT (**p < 0.01 vs. the saline group) and chronic nicotine treatment significantly alleviated this reduction (##*p* < 0.01 vs. the LPS group). (C) Neither LPS nor chronic nicotine had significant differences in the win‐shift failure in the DAT test (*p* > 0.05). (D) LPS increased the lose‐shift failure of mice in the DAT test (**p < 0.01 vs. the saline group) and chronic nicotine markedly inhibited such effect (#*p* < 0.05 vs. the LPS group). (E) Neither LPS nor nicotine had a significant effect on the motor function of the mice (*p* > 0.05; *n* = 11).

This test aimed to examine the impact of 2‐week nicotine treatment on LPS‐produced WM deficits. Mice were randomly assigned into four groups: (1) saline group, (2) LPS group (0.5 mg/kg, i.p.), (3) (0.2 mg/kg, s.c.) + LPS (0.5 mg/kg, i.p.), and (4) nicotine group (0.2 mg/kg, s.c.) (see Figure 2A for detailed experimental procedures).

##### Experimental 3

This test aimed to examine the effect of blocking the IL‐1 receptor on LPS‐produced WM deficiency. Mice were randomly assigned into saline, LPS (0.5 mg/kg, i.p.), IL‐1Ra (1 μg/1 μL/day, intra‐PFC) + LPS (0.5 mg/kg, i.p.), and IL‐1Ra groups (1 μg/1 μL/day, intra‐PFC) (see Figure  5E for detailed experimental procedures).

**FIGURE 3:**
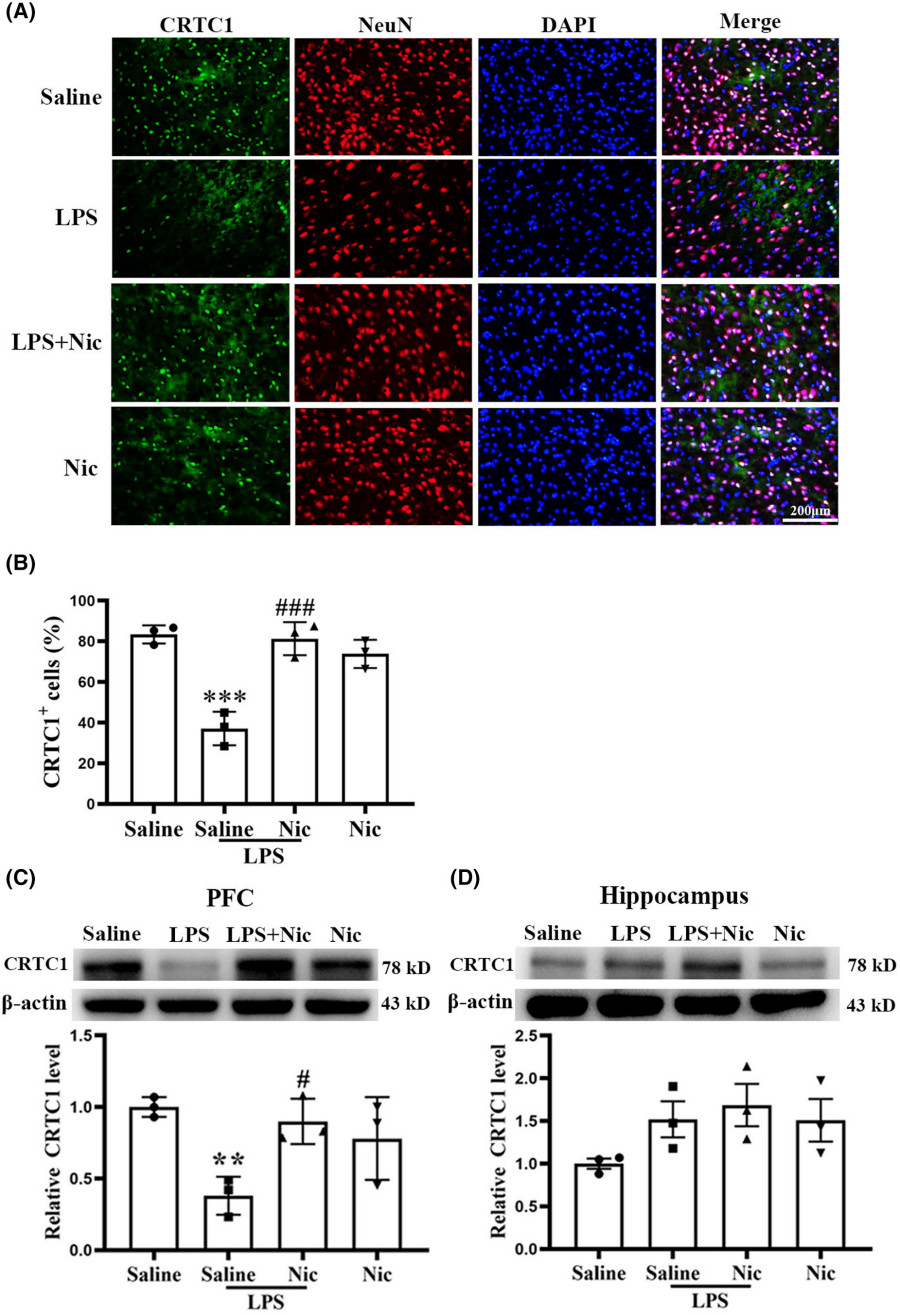
Chronic nicotine treatment significantly inhibited LPS‐induced CRTC1 reduction in PFC. (A, B) Immunofluorescence staining indicated that LPS markedly reduced the expression level of CRTC1 (green) in the PFC (***p < 0.001 vs. the saline group, *n* = 3) and nicotine treatment can markedly alleviate such effect (^###^ *p* < 0.001 vs. the LPS group; *n* = 3; scale bar = 200 μm). (C) WB results demonstrated that LPS significantly reduced the level of CRTC1 in the PFC (**p < 0.01 vs. the saline group, *n* = 3) and chronic nicotine markedly inhibited such effect (^#^ *p* < 0.05 vs. the LPS group, *n* = 3). (D) Western blot results demonstrated that neither LPS nor nicotine significantly affected the expression of CRTC1 in hippocampus (*p* > 0.05; *n* = 3).

##### Experimental 4

This experiment aimed to examine the impact of blocking the HCN channel on WM. Mice were divided into three groups randomly: (1) saline group, (2) ZD7288 (1.5 μg/μL), and (3) ZD7288 (15 μg/μL). After perfusion with PBS/PFA, the brain tissue was removed and preserved for subsequent experimental analysis (see Figure 6A for detailed experimental procedures).

### 2.5 Immunoblotting for CRTC1 and HCN2

Mice received overdose anesthesia with pentobarbital and were perfused with precooling PBS after the behavioral test. The expression levels of HCN2 and CRTC1 in the mouse hippocampus and mPFC were analyzed. Tissues were homogenized and lysed in a lysis buffer (50 mM Tris–HCl, 150 mM NaCl, 5 mM CaCl_2_·2H_2_O, 1% triton X‐100, and 0.05% Brij‐35, pH = 7.6). The protein concentration was measured using a BCA protein assay kit (Beyotime, Haimen, China). Based on our published paper previously,^29^ equal amounts (30 μg of total protein) of each homogenate aliquot were boiled at 100°C for 5 min and then the proteins were separated by electrophoresed in 10% or 12.5% SDS‐PAGE gels, transferred onto PVDF membranes (0.45 μm, Millipore, Billenca, USA). Membranes were subsequently blocked within TBS‐T (Tris buffer saline and 0.1% Tween‐20) containing 5% nonfat milk for 1 h, and incubated with primary antibodies CRTC1 (1:1000, Cell Signaling Technology, USA), HCN2 (1:1000, Alomone USA), or β‐actin (1:5000, Boster, China) at 4°C for 12 h. After TBS‐T washing, the membranes were incubated with the corresponding secondary antibodies (Boster, China) for 2 h. Finally, the membranes were subjected to incubation with the SuperSignal West Pico HRP substrate reagents (Thermo Fisher, USA) and imaging capture. The protein expression levels of HCN2 and CRTC1 were quantified using Image J software and normalized with β‐actin as the loading control.

### 2.6 Immunofluorescence staining for HCN2 and CRTC1

Immediately after the behavioral test, mice received overdose anesthesia with pentobarbital and were subsequently perfused with PBS and fixed by 4% PFA. The brain tissues were then sectioned into 20‐μm‐thick cryosections for the evaluation of HCN2 and CRTC1, as previously described.^30^ Briefly, nonspecific component was removed with a blocking buffer containing 0.3% Triton X‐100, 1% BSA, and 5% goat serum. The blocked sections were incubated first with primary antibodies against CRTC1 (1:200, Cell Signaling Technology, USA), HCN2 (1:200, Alomone), or NeuN (1:500, Millipore) overnight at 4°C, and then with the secondary antibody (anti‐rabbit Alexa Fluor 488 conjugated, diluted at 1:800) for 1 h at room temperature. The staining of mPFC sections was captured using a Zeiss LSM 700 microscope.

### 2.7 ELISA to detect TNF‐α and IL‐1β

Mice received overdose anesthesia with pentobarbital and were perfused with PBS immediately after the behavioral test. The medial prefrontal cortex (mPFC) was collected for ELISA (Boster, China). Tissues were homogenized, lysed, and quantified. The total protein concentration of the brain homogenates was adjusted to 1 mg/mL, and the concentrations of TNF‐α and IL‐1β in the samples were determined according to the kit instructions. Briefly, the protein supernatant, containing potential TNF‐α and IL‐1β, was incubated at 37°C with the coated antibody 1.5 h and then corresponding antibody for 1 h in 96‐well plates, respectively. After washing, avidin–biotin–peroxidase complex (ABC) was added and incubated for 30 min at 37°C. Following another washing step, tetramethylbenzidine (TMB) substrate was added and incubated for 15 min at 37°C. The absorbance of each well was measured at 450 nm, and the concentrations of TNF‐α and IL‐1β were determined using a standard curve.

### 2.8 Real‐time RT‐PCR

Total RNA was extracted from brain tissue using the TRIzol reagent (Invitrogen, USA) according to the manufacturer's instructions. cDNA synthesis was then performed with 3 μg of total RNA and random primers using TaqMan® Reverse Transcription Kits (Applied Biosystems) in a 20 μL reaction volume. The reaction mixture was incubated at the appropriate temperature and time as specified in the kit protocol to complete reverse transcription. The quantification primers (synthesized by Integrated DNA Technologies) used in this study were designed according to the known mouse sequences: IL‐1β forward: 5′‐TGGAAAAGCGGTTTGTCTTC‐3′, reverse: 5′‐TACCAGTTGGGGAACTCTGC; TNF‐α forward: 5′‐CATCTTCTCAAAATTCGAGTGACAA‐3′, reverse: 3′‐TGGGAGTAGACAAGGTACAACCC‐5′; GAPDH forward: 5′‐CAATGTGTCCGTCGTGGATCT‐3′, reverse: 3′‐GTCCTCAGTGTAGCCCAAGATG‐5′. The SDS Enterprise Database software (Applied Biosystems) was employed for computing the fluorescence threshold cycle (Ct) value. Fold changes in mRNA expression were determined by ΔΔCt, as previously described,^13^, ^31^ and expressed as $2^{-\Delta \Delta C_{t}}$ values.

### 2.9 Statistical analysis

Formal tests for normality were used to assess data distribution. Data are shown as the means ± SEM.

Statistical analysis was performed using the SPSS 18.0 software (SPSS, Chicago, IL, USA). Either one‐way ANOVA or two‐way ANOVA, followed by Bonferroni post hoc tests, was used for data analysis. *p* < 0.05 was considered statistically significant.

## 3 RESULTS

### 3.1 Effect of acute nicotine on LPS‐induced WM impairment in DAT task

LPS (0.5 mg/kg) treatment significantly impaired mice WM in the DAT task,^7^ and acute nicotine (0.2 mg/kg) treatment can improve rats' spatial WM in the DAT task.^25^ Using DAT task, we detected the impact of acute nicotine treatment on LPS‐produced WM deficit in DAT task in mice. Figure 1A shows the experimental procedure of acute nicotine treatment. As shown in Figure 1B, LPS treatment significantly reduced the accuracy rate of mice in the DAT task (*F*(1, 16) = 22.086, *p* < 0.01). Acute nicotine treatment failed to significantly improve the LPS‐induced decrease in correct choice (*F*(1, 16) = 1.807, *p* > 0.05). As shown in Figure 1C, neither LPS nor nicotine treatment had significant effect on win‐shift‐failure (*F*(1, 17) = 3.821, *p* > 0.05), (*F*(1, 17) = 1.391, *p* > 0.05). As demonstrated in Figure 1D, administration of LPS resulted in a significant increase in lose‐shift‐failure (*F*(1, 17) = 10.321, *p* < 0.01). Acute nicotine treatment did not lead to a reduction in the LPS‐induced upregulation of lose‐shift‐failure (*F*(1, 17) = 0.024, *p* > 0.05). As shown in Figure 1E, neither LPS (*F*(1, 17) = 4.807, *p* > 0.05) nor acute nicotine (*F*(1, 17) = 2.248, *p* > 0.05) treatment did alter the motor function. The above results show that mice treated with LPS significantly impaired the WM, while acute nicotine treatment did not significantly affect LPS‐induced WM deficiency.

### 3.2 Effect of chronic nicotine on LPS‐induced WM deficit in DAT task

Chronic nicotine can selectively improve short‐term memory in the G72 schizophrenia mouse model.^32^ In this study, Here, we examined the effect of chronic treatment with nicotine on LPS‐produced WM deficiency. The detailed experimental procedures are shown in Figure 2A, and systemic LPS treatment significantly impaired mice performance in the T‐maze task (Figure 2B). A two‐way ANOVA revealed a main effect of LPS (*F*(1, 52) = 4.013, *p* < 0.05), as did nicotine (*F*(1, 52) = 4.045, *p* < 0.05), and there was a notable interaction between LPS and nicotine (*F*(1, 52) = 7.691, *p* < 0.01), indicating that nicotine treatment alleviated LPS‐induced decrease in correct choice. More interestingly, analysis of error types revealed that LPS interference affected both the win‐shift and lose‐shift strategies. Interestingly, administration of nicotine did not enhance the utilization of the win‐shift strategy (Figure 2C) as there is no main effect of LPS (*F*(1, 52) = 0.32, *p* > 0.05), no main effect of nicotine (*F*(1, 52) = 2.672, *p* > 0.05), and no interaction of LPS × nicotine (*F*(1, 52) = 3.222, *p* > 0.05). A two‐way ANOVA analysis on lose‐shift failure (Figure 2D) demonstrated a significant main effect of LPS (*F*(1, 52) = 11.251, *p* < 0.01), interaction of LPS × nicotine (*F*(1, 52) = 7.806, *p* < 0.01), and main effect of nicotine (*F*(1, 52) = 4.448, *p* < 0.01). This implies that nicotine treatment‐produced improvement in WM may be attributed to enhanced proficiency in employing the lose‐shift strategy. There were no significant alterations in mice motor activity following either LPS or nicotine treatment (Figure 2E), as indicated by a two‐way ANOVA showing no main effect of LPS (*F*(1, 52) = 3.777, *p* > 0.05), nicotine (*F*(1, 52) = 0.007, *p* > 0.05), and no interaction between LPS and nicotine (*F*(1, 52) = 0.229, *p* > 0.05). Therefore, the performance deficit in the T‐maze during the test was not owing to the effect on motor activity (Figure 2E).

### 3.3 Pretreatment with nicotine reduced the LPS‐induced decrease in CRTC1

Double‐immunofluorescence staining of NeuN and CRTC1 displayed the expression of CRTC1 in PFC. As shown in Figure 3A, CRTC1 was localized at neurons in mPFC. Quantification results showed that pretreatment with nicotine reduced LPS‐induced decrease in CRTC1 (Figure 3B). Western blot results confirmed the CRTC1 expression changes in PFC as a two‐way ANOVA on CRTC1 level in PFC (Figure 3C) revealed a significant main effect of LPS (*F*(1, 11) = 15.717, *p* < 0.01), the interaction of LPS × nicotine (*F*(1, 11) = 12.51, *p* < 0.05), but no main effect of nicotine (*F*(1, 11) = 2.047, *p* > 0.05). In addition, western blot results showed that treatment with neither LPS nor nicotine significantly changed CRTC1 expression in hippocampus, as a two‐way ANOVA on CRTC1 level in hippocampus (Figure 3D) showed no main effect of LPS (*F*(1, 11) = 2.849, *p* > 0.05), no main effect of nicotine (*F*(1, 11) = 2.672, *p* > 0.05), no interaction of LPS × nicotine (*F*(1, 11) = 0.685, *p* > 0.05).

### 3.4 Pretreatment with nicotine reduced LPS‐induced decrease in HCN2

**FIGURE 4:**
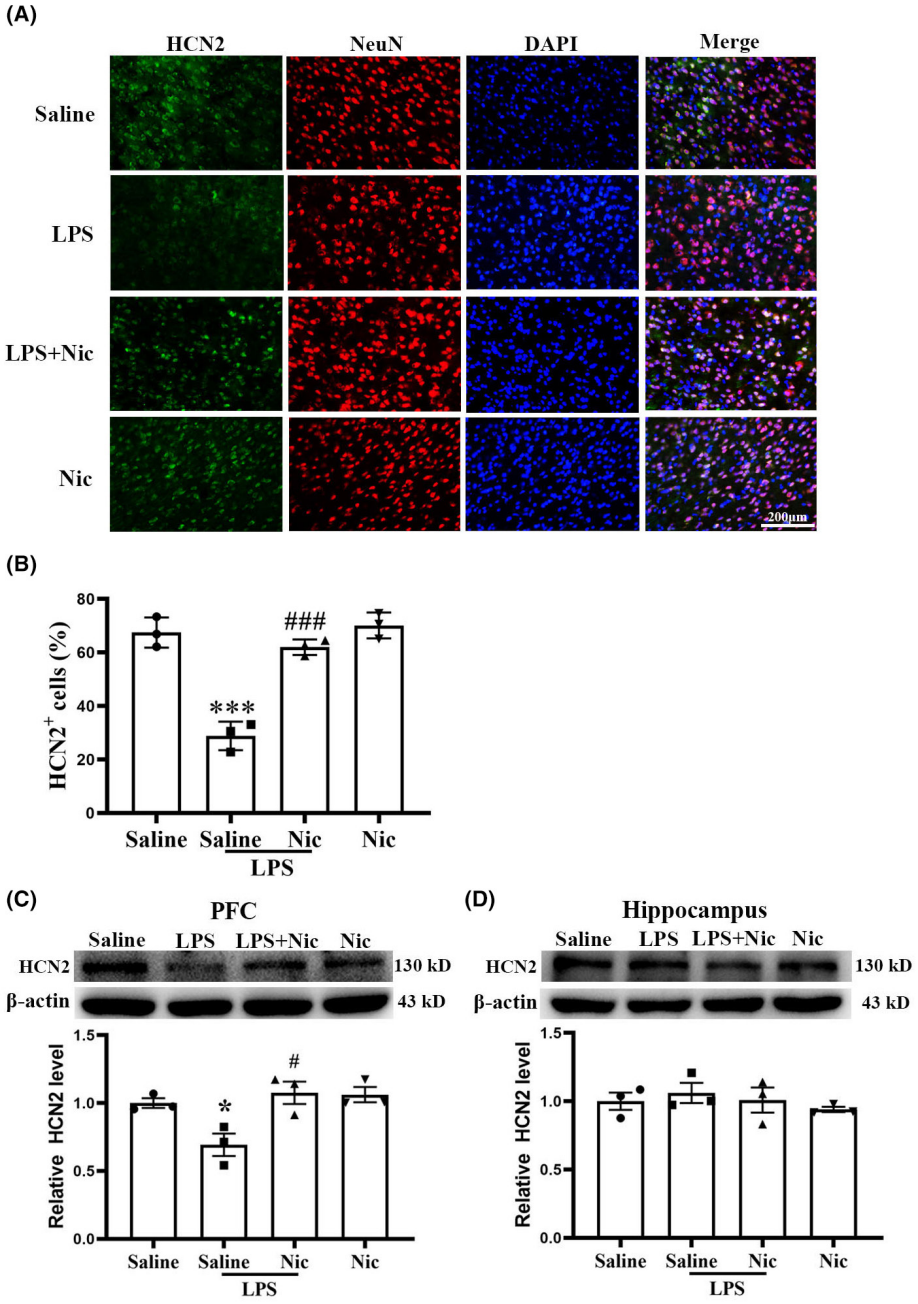
Chronic nicotine treatment significantly inhibited LPS‐induced HCN2 reduction in PFC. (A, B) Immunofluorescence results showed that HCN2 (green) and NeuN (red) are co‐localized and LPS significantly reduced the expression of HCN2 in the PFC (***p < 0.001 vs. the saline group, *n* = 3), and chronic nicotine treatment markedly inhibited such effect (^###^ *p* < 0.001 vs. the LPS group; *n* = 3; scale bar = 200 μm). (C) Western blot results showed that LPS significantly reduced level of the HCN2 in the PFC (*p < 0.05 vs. the saline group, *n* = 6), and chronic nicotine significantly inhibited such effect (^#^ *p* < 0.05 vs. the saline group; *n* = 6). (D) Western blot results demonstrated that neither LPS nor nicotine significantly affected the expression of HCN2 in hippocampus (*p* > 0.05; *n* = 3).

HCN channels are crucial in the formation of working memory^20^ and upregulating the expression of HCN2 in the PFC of rats inhibited WM deficiency caused by bilateral common carotid artery occlusion.^17^ Co‐staining of NeuN and HCN2 was done to show the distribution of HCN2 in mPFC. As depicted in Figure 4A, HCN2 was found in neurons in mPFC, and LPS treatment notably downregulated the expression of HCN2 in mPFC, and nicotine pretreatment significantly ameliorated such reduction (Figure 4B). Western blot results confirmed the HCN2 expression change in PFC (Figure 4C), and a two‐way ANOVA showed a main effect of LPS (*F*(1, 11) = 6.772, *p* < 0.05), a main effect of nicotine (*F*(1, 11) = 8.999, *p* < 0.05), and a main effect of interaction of LPS × nicotine (*F*(1, 11) = 5.733, *p* < 0.05). As shown in Figure 4D, treatment with neither LPS nor nicotine significantly changed the HCN2 expression in hippocampus, and a two‐way ANOVA on HCN2 level in hippocampus showed no main effect of LPS (*F*(1, 11) = 0.923, *p* > 0.05), no main effect of nicotine (*F*(1, 11) = 0.685, *p* > 0.05), and no interaction of LPS × nicotine (*F*(1, 11) = 0.003, *p* > 0.05).

### 3.5 Pretreatment with nicotine significantly reduced LPS‐induced mRNA and protein upregulation of IL‐1β and TNF‐α in the medial prefrontal cortex (mPFC)

**FIGURE 5:**
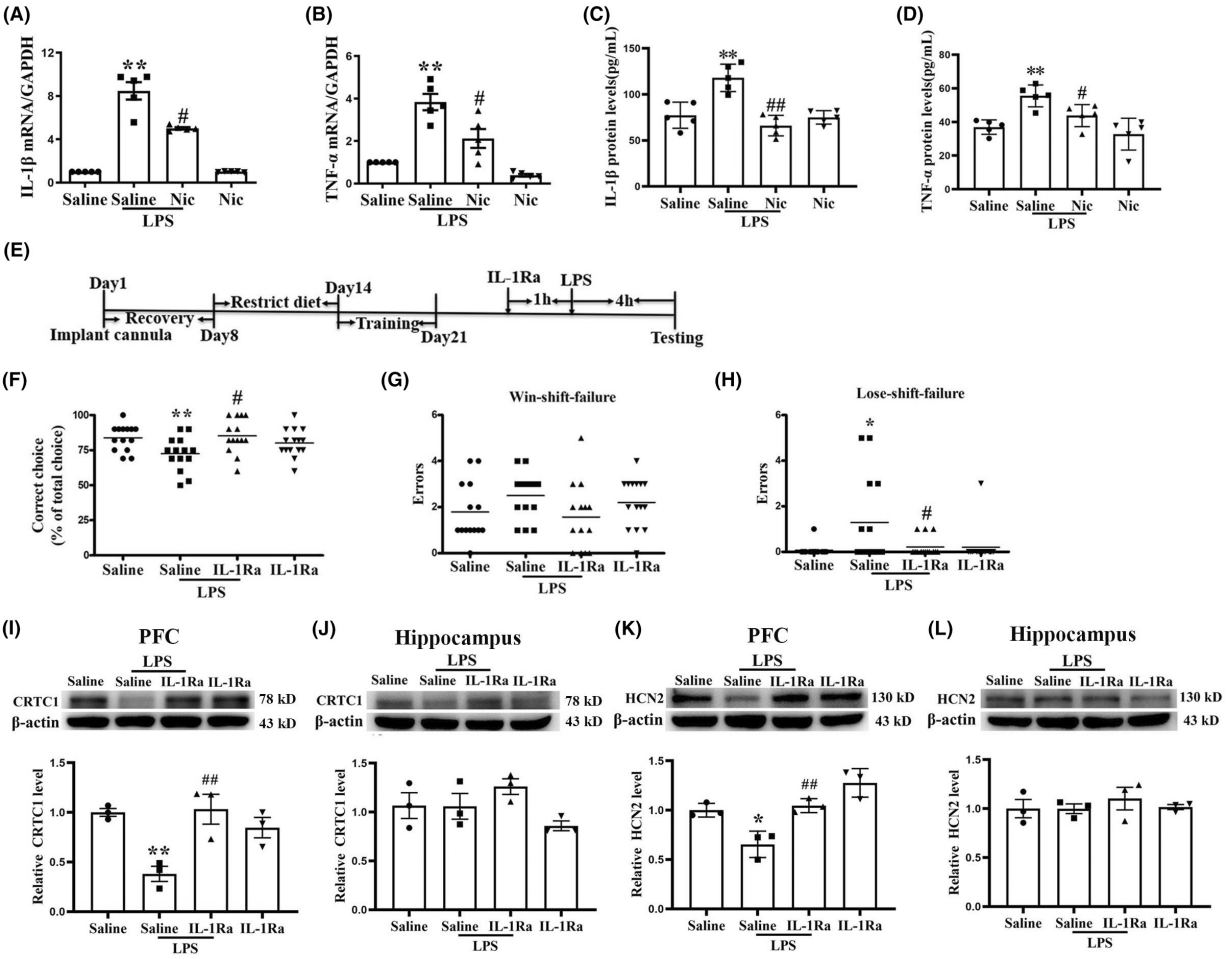
Pretreatment with nicotine significantly reduced LPS‐induced IL‐1β and TNF‐α increase in PFC and blockade of IL‐1 β alleviated LPS‐induced WM deficit in the DAT task. LPS induced a significant effect on increasing the mRNA levels of pro‐inflammatory factors IL‐1β (A) and TNF‐α (B) in the PFC (**p < 0.01 vs. the saline group; *n* = 5), and chronic nicotine treatment significantly inhibited such effect (^#^ *p* < 0.05 vs. the LPS group; *n* = 5). The protein levels of pro‐inflammatory factors IL‐1β (C) and TNF‐α (D) in the PFC were significantly increased by LPS treatment (**p < 0.01 vs. the saline group; *n* = 5), and chronic nicotine treatment markedly alleviated such effect (^#^ *p* < 0.05, ^##^ *p* < 0.01 vs. the LPS group; *n* = 5). (E) Experimental procedure. (F) LPS significantly reduced the correct choice rate of mice in the DAT task (**p < 0.01 vs. the saline group, *n* = 14), and IL‐1Ra significantly suppressed such reduction (^#^ *p* < 0.05 vs. the LPS group; *n* = 14). (G) Neither LPS nor IL‐1Ra significantly affected the win‐shift failure (*p* > 0.05; *n* = 14). (H) LPS markedly increased the lose‐shift failure (*p < 0.05 vs. the saline group, *n* = 14), and IL‐1Ra significantly alleviated such effect (^#^ *p* < 0.05 vs. the LPS group; *n* = 14). (I) Western blot results demonstrate that LPS significantly downregulated the level of CRTC1 expression in the PFC (**p < 0.01 vs. the saline group, *n* = 3) and IL‐1Ra significantly suppressed such effect (^##^ *p* < 0.01 vs. the LPS group, *n* = 3). (J) Western blot results demonstrated that neither LPS nor IL‐1Ra significantly affect the expression of CRTC1 in hippocampus (*p* > 0.05; *n* = 3). (K) Western blot results demonstrated that LPS significantly reduced level of the HCN2 in the PFC (*p < 0.05 vs. the saline group, *n* = 6), and IL‐1Ra had a significant effect on inhibiting such effect (^##^ *p* < 0.01 vs. the saline group; *n* = 6). (L) Western blot results demonstrated that neither LPS nor IL‐1Ra significantly affected the expression of HCN2 in hippocampus (*p* > 0.05; *n* = 3).

LPS can impair WM in rats^33^ and D‐galactose‐impaired rat's WM and decreased spontaneous alternation in the Y‐maze by increasing TNF‐α and IL‐1β.^34^ Here, we detected the mRNA (Figure 5A,B) and protein (Figure 5C,D) levels of IL‐1β and TNF‐α. As can be seen in Figure 5A,B, mice treated with LPS significantly increased the mRNA level of IL‐1β (Figure 5A) and TNF‐α (Figure 5B) in the PFC, and 2‐week nicotine treatment significantly reduced the upregulation. In addition, ELISA results showed that LPS treatment upregulated the protein of IL‐1β (Figure 5C) and TNF‐α expression (Figure 5D) in PFC, and 2‐week nicotine treatment inhibited such increase. The above experimental results indicate that nicotine treatment may improve LPS‐induced WM deficiency by decreasing TNF‐α and IL‐1β in mPFC.

### 3.6 Effect of blockade IL‐1 β on LPS‐induced WM impairment in DAT task

We next explored whether blocking IL‐1β could reduce LPS‐induced WM deficiency. The detailed experimental procedures are shown in Figure 5E. Figure 5F shows that mice treated with LPS significantly reduced the accuracy, and intra‐mPFC‐infused antagonist IL‐1Ra markedly increased the accuracy of mice in DAT and a two‐way ANOVA revealed a main effect of interaction of LPS × IL‐1Ra (*F*(1, 55) = 7.319, *p* < 0.01), but no main effect of LPS (*F*(1, 55) = 1.469, *p* > 0.05) and no main effect of IL‐1Ra (*F*(1, 55) = 2.895, *p* > 0.05), suggesting that IL‐1Ra treatment significantly ameliorated systemic LPS‐impaired correct choice in mice. More interesting, analysis of the error types showed that systemic LPS treatment disrupted lose‐shift but not win‐shift strategy and IL‐1Ra treatment did not improve the ability to apply win‐shift strategy (Figure 5G) as there is no main effect of LPS (*F*(1, 55) = 0.113, *p* > 0.05) and IL‐1Ra (*F*(1, 55) = 1.013, *p* > 0.05), and no interaction of IL‐1Ra × LPS (*F*(1, 55) = 3.616, *p* > 0.05). A two‐way ANOVA analysis on lose‐shift failure (Figure 5H) demonstrated a main effect of LPS (*F*(1, 55) = 4.587, *p* < 0.05), interaction of LPS × IL‐1Ra (*F*(1, 55) = 4.587, *p* < 0.05), but no main effect of IL‐1Ra (*F*(1, 55) = 2.683, *p* > 0.05), suggesting that IL‐1Ra treatment ameliorated systemic LPS‐produced WM deficiency by increasing the ability to apply lose‐shift strategy. Western blot results showed that IL‐1Ra treatment significantly inhibited LPS‐induced CRTC1 and HCN2 downregulation in mPFC (Figure 5I,K). In contrast, treatment with neither LPS nor IL‐1R significantly altered the expression of CRTC1 and HCN2 in hippocampus (Figure 5J,L).

### 3.7 Effect of blocking HCN channel on working memory (WM) in DAT task

**FIGURE 6:**
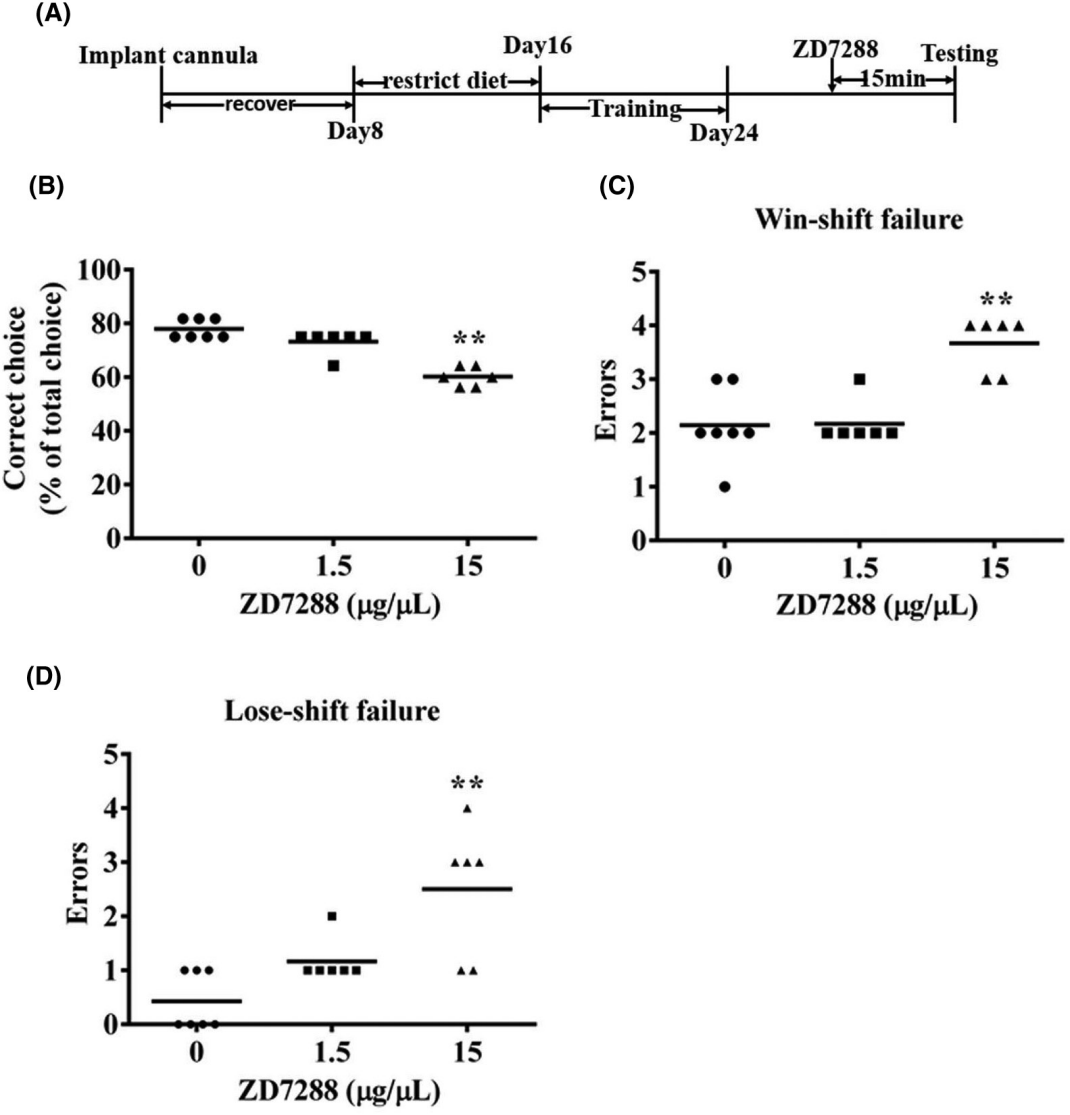
Effect of HCN2 block on working memory (WM) in DAT task. Bilateral microinjection of 15 μg/μL HCN channel blocker ZD7288 into the PFC significantly caused WM deficiency in the DAT in mice. (A) Experimental flow chart. (B) 15 μg/μL but not 1.5 μg/μL ZD7288 significantly decreased the correct choice of mice in DAT (**p < 0.01). (C) 15 μg/μL but not 1.5 μg/μL ZD7288 significantly increased the win‐shift failure (**p < 0.01). (D) 15 μg/μL but not 1.5 μg/μL ZD7288 significantly increased the lose‐shift failure (**p < 0.01 vs. the saline group; *n* = 6–7).

ZD7288, a blocker of HCN channel, impaired hippocampus‐dependent memory.^28^ Here, we tested if blocking HCN channel could disrupt WM. Mice received bilateral intra‐PFC injection of ZD7288, which dose‐dependently disrupted WM. One‐way ANOVA showed that ZD7288 significantly decreased correct choice (*F*(2, 17) = 35.58, *p* < 0.05, Figure 6B), and increased win‐shift failure (*F*(2, 17) = 14.96, *p* < 0.05, Figure 6C) and lose‐shift failure (*F*(2, 17) = 11.19, *p* < 0.05 Figure 6D). Bonferroni post hoc analysis showed that 15 μg/μL, but not 1.5 μg/μL ZD7288, resulted in a significant deficiency in WM in the DAT in T‐maze.

## 4 DISCUSSION

**FIGURE 7:**
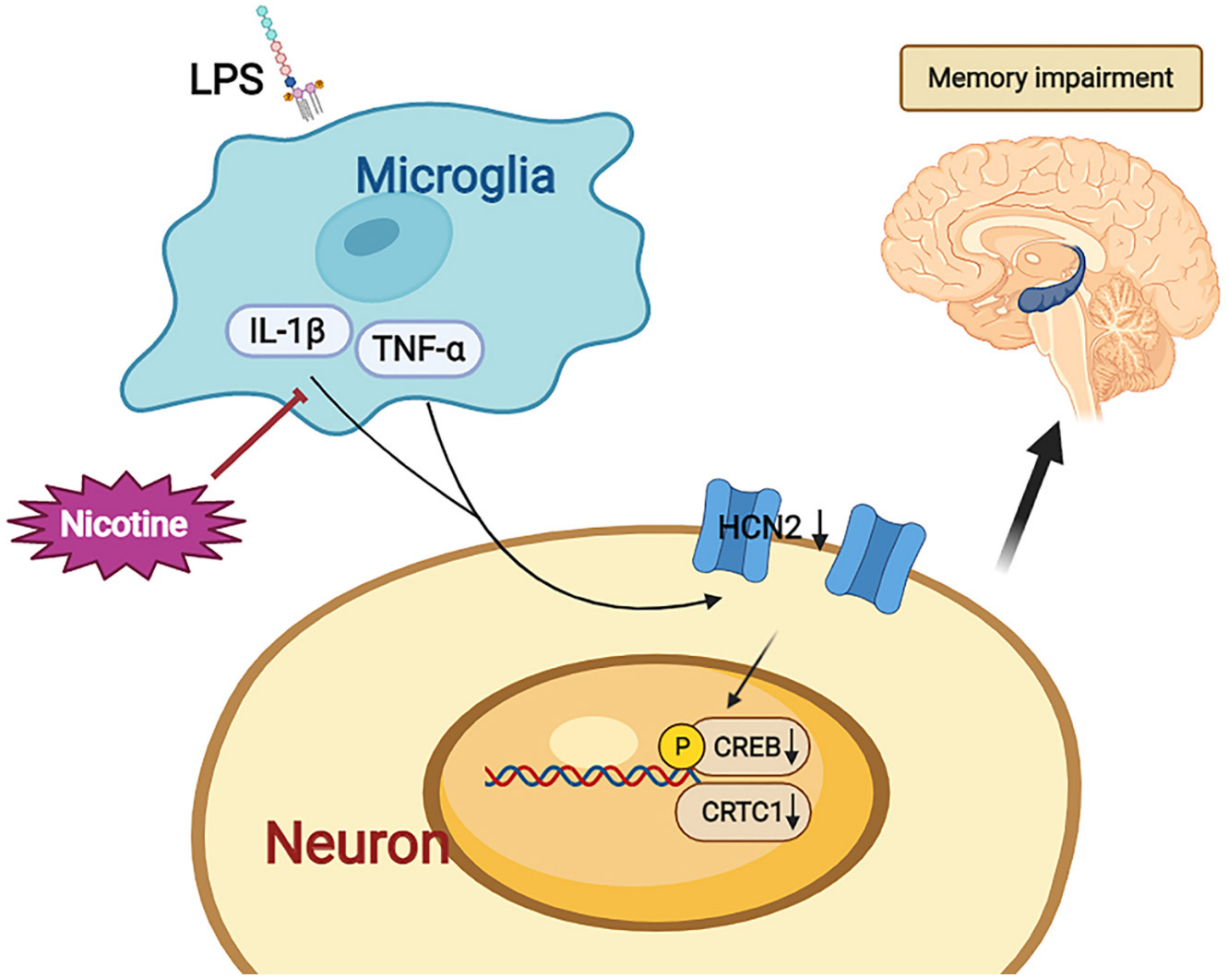
Summary. LPS significantly induced WM deficit in mPFC, accompanied by IL‐1β and TNF‐α upregulation. Nicotine treatment significantly alleviated LPS‐induced WM deficit by upregulating CRTC1 and HCN2.

In this study, we demonstrated that 2 weeks but not acute nicotine treatment significantly ameliorated LPS‐produced WM deficiency. Specifically, the treatment enhanced the ability to apply the lose‐shift strategy, but not the win‐shift strategy. We demonstrated that LPS treatment significantly produced WM deficiency accompanied by the upregulation of both IL‐1β and TNF‐α in mPFC. Additionally, intra‐mPFC infusion of the IL‐1 antagonist IL‐1Ra markedly alleviated LPS‐induced WM deficiency. More importantly, chronic but not acute nicotine treatment had a significant effect on alleviating LPS‐induced WM deficit by upregulating CRTC1 and HCN2 (Figure 7). Of note, intra‐mPFC infusion of HCN channel blocker ZD7288 induced significant WM deficiency.

The T‐maze is used to detect spatial working memory, in which animals inherently tend to switch arm exploration, that is, spontaneous alternation during successive experiments. While the selection of the target arm is based on remembering the last explored target arm, that is, spatial working memory, therefore, the correct alternate choice of the target arm by the animal is a manifestation of the ability of spatial working memory.^35^, ^36^ The T‐shaped maze fully utilizes the foraging nature of animal exploration, which can minimize the factors that affect the experimental results, and compared with other detection methods, the T‐shaped maze is easier to detect.^37^

Our previous results showed that acute nicotine (salt, 0.2 mg/kg, s.c.) ameliorated WM in DAT task in male rats,^25^ but in the current study, acute nicotine treatment at the same dose did not significantly alleviate LPS‐induced WM deficit (Figure 1). Improvement of WM under physiological and pathological conditions is very different and maybe higher dose or combination treatment is required for acute nicotine treatment to improve LPS‐induced WM deficit. In addition, Wei et al. have shown that acute nicotine treatment reduced LPS‐produced cognitive deficit through upregulating BDNF expression and decreasing neuroinflammation in rat hippocampus.^38^ The difference may be dependent on the complexity of the behavior task and nicotine dose; in Wei's study, they used water maze task to detect spatial memory, whereas in our study, we used DAT T‐maze task to detect WM which includes both the hippocampus‐dependent spatial working memory and PFC‐dependent ability to correct error. In addition, they used a higher dose of nicotine, 0.5 mg/kg versus the dosage, used in this study (0.2 mg/kg).

We demonstrated that chronic nicotine treatment could significantly improve LPS‐produced WM deficiency (Figure 2). Nicotine treatment has been reported to ameliorate maternal LPS exposure‐induced schizophrenia‐like cognitive deficits which were detected by prepulse inhibition, latent inhibition, and delayed nonmatching to sample.^39^ In addition, in the G72 schizophrenia mouse model, chronic nicotine treatment has been shown to selectively improve short‐term memory.^32^ Chronic treatment could mimic the smoking status because it is reported that the nicotinic receptor has a critical role in cigarette smoking‐induced improvement in schizophrenia‐associated spatial working memory and attentional deficits.^40^

It has been reported that α7 nAchRs in PFC play important roles in both WM and reference memory, while α4β2 nAchRs only play critical roles in WM.^41^ In addition, in spatial learning and memory, β2‐containing receptors are key to maintaining normal sex differences, while in radial‐arm maze learning, α7 nAchRs appear to be critical for cognitive function independent of sex.^42^

Aligning with our previous study,^7^ we showed that acute LPS treatment impaired WM in the DAT task. Notably, we also provided evidence showing that LPS could significantly increase IL‐1β and TNF‐α levels in PFC, suggesting that LPS‐induced WM may be mediated by neuroinflammation which is known to play a key role in the impairment of fear memory.^13^ LPS impaired WM by inducing pro‐inflammatory cytokines, including IL‐1β, IL‐6, and TNF‐α, which can directly impair memory in passive avoidance task.^43^ Furthermore, a previous study showing a significant upregulation of IL‐1β in mPFC but not hippocampus 4 h after LPS treatment^44^ suggests that LPS‐induced IL‐1β increase could be critical for LPS‐induced WM deficiency. Here, we demonstrated that intra‐PFC infusion of IL‐1 antagonist IL‐1Ra blocked LPS‐induced WM deficiency (Figure 5), providing evidence showing that LPS‐induced WM was mediated by IL‐1β, and nicotine may improve WM through modulating IL‐1β levels.

HCNs are critically implicated in WM,^20^ and here we showed that blocking the HCN channel impaired the WM (Figure 6), suggesting that LPS may impair the WM by regulating the activity of HCN2. Chronic cerebral hypoperfusion has been shown to impair spatial working memory by downregulation of HCN2 expression in the PFC in rats.^17^, ^18^ In the present study, the results revealed that LPS significantly decreased the expression of HCN2 in PFC, and chronic nicotine could alleviate this impairment by upregulating the expression of HCN2 (Figure 4), suggesting that HCN2 could be a target for reducing LPS‐produced WM deficiency.

We demonstrated LPS‐produced CRTC1 decrease in PFC (Figure 3) which is known to be crucial for fear memory consolidation,^9^ ischemic stroke‐produced memory deficit,^11^ and schizophrenia‐induced WM deficiency.^45^ We have shown that LPS impaired fear memory reconsolidation through downregulating CRTC1 expression.^13^ Our current study indicates that CRTC1 is also involved in WM and could be a target for reducing LPS‐produced WM deficiency.

We demonstrated that LPS treatment downregulated the expression of HCN2 and CRTC1 in the mPFC but not in the hippocampus, and that chronic nicotine treatment alleviated such changes associated with a decrease in lose‐shift failure, indicating that the decreased HCN2 and CRTC1 in the mPFC played crucial roles in the induction of lose‐shift failure by LPS. Since win‐shift failure indicates deficiency in WM ability (the retention of short‐term information), and lose‐shift failure implies a disability of correcting errors,^25^, ^46^, ^47^ our study indicated that PFC played a more critical role in LPS‐induced WM deficiency.

### 4.1 Limitations of this study

Only male mice were used in the study and only one model was used to assess spatial memory. In addition, testing was done only at one time point, thus it is not known if spatial working memory impairments are transitory or long lasting. Furthermore, the precise mechanism of how nicotine alters HCN2 and CRCT1 expression is not fully understood, and needs to be explored further.

## 5 CONCLUSIONS

Our results demonstrate that acute exposure to LPS in male mice produced WM impairment, characterized by decreased expression of CRTC1 and HCN2 in the mPFC, but not in the hippocampus. Notably, chronic nicotine treatment significantly improved LPS‐induced WM deficits by enhancing the ability to utilize the lose‐shift strategy and upregulating the expression of CRTC1 and HCN2 within the mPFC.

## AUTHOR CONTRIBUTIONS

X Wang, Q Wang, M Song, Y Wang, X Shen, and Y Sun contributed to the execution of the entire research project and the statistical analyses. X Wang, Q Wang, and P Geng drafted the manuscript. C Guo revised the manuscript. C Ma and X Jin designed the experiment, supervised the project, and finalized the manuscript.

## CONFLICT OF INTEREST STATEMENT

The authors declare that they have no conflicts of interest.

## Data Availability

The data that support the findings of this study are available from the corresponding author upon reasonable request.
